# Assessing avian diversity and red squirrel occurrence in fragmented high-altitude mountain pine forests of the central French Pyrenees: A dataset of point counts

**DOI:** 10.1016/j.dib.2024.110660

**Published:** 2024-06-21

**Authors:** Michel Génard, Françoise Lescourret

**Affiliations:** INRAE, UR PSH, Avignon 84000, France

**Keywords:** Bird, Squirrel, Mountain, Point-count, Pine forest, Fragmentation, Habitat, Landscape

## Abstract

In the spring of 1987, point-count surveys of breeding birds (passerines and picidae) were conducted, resulting in a dataset of 197 counts. The purpose was to analyze the effects of forest fragmentation on bird community composition in a mountain pine forest located in the Néouvielle National Nature Reserve in the central French Pyrenees between 1800 and 2400 metres. The study aimed to differentiate between the impacts of landscape factors (patch area, isolation) and habitat characteristics (altitude, vegetation structure). Additional information was gathered regarding the presence of Common Crossbill (Loxia curvirostra), Great Spotted Woodpecker (Dendrocopos major), Red Squirrel (Sciurus vulgaris), and Capercaillie (Tetrao urogallus) in the forest. The sampling design ensured that the selected patches represented a wide range of sizes and distances to the nearest large pine patch or low-altitude forest stand. Bird sampling utilized the point-count technique [3], focusing on singing passerines and Picidae within a 50-metre radius. The altitude, the percentage of open areas, of stones, boulders and of herbaceous and ligneous plant cover at various heights, the canopy height and number of dead trees, along with landscape variables describing patch size and isolation from large pine stands or low-altitude forests, were assessed for each point count. This dataset offers insight into the breeding bird community and squirrel occurrence in a typical high-altitude mountain pine forest in the Pyrenees in 1987, serving as a baseline for future comparisons to study changes in bird and squirrel populations, the impact of climate change, habitat fragmentation, and conservation priorities. These data aim to inspire further research and enhance our understanding of bird and squirrel ecology in mountain regions*.*

Specifications Table

**Table:** 

Subject	Biology
Specific subject area	Biodiversity
Type of data	Table
Data collection	20 min bird point-counts were conducted in the morning in spring within a distance of 50 metres around the observer. The presence of pine cones eaten by Common Crossbill, Great Spotted Woodpecker, Red Squirrel and Capercaillie droppings were also recorded at each point count. Within a 50-metre radius of each point count, the altitude, the percentage cover of open areas, of stones and boulders and of various vegetation layers, were estimated, along with the canopy height and the number of dead trees. The landscape variables, which included information on patch size and isolation from large pine stands or low-altitude forests, were determined using IGN maps and aerial photographs.
Data source location	Néouvielle National Nature Reserve in the central French Pyrenees, France N 42°47′ 15.3594‘’ - 42°53′ 45.9594′', E 0°3′ 32.7594‘’ - 0°14′ 16.44′' (Fig. 1)
Data accessibility	Repository name: Recherche Data Gouv https://entrepot.recherche.data.gouv.fr/dataverse/inrae Data identification number: doi: 10.57745/AMIZQK Direct URL to data: https://entrepot.recherche.data.gouv.fr/dataset.xhtml?persistentId=doi:10.57745/AMIZQK
Related research article	Lescourret F., Génard M. 1994. Habitat, landscape and bird composition in mountain forest fragments. Journal of Environmental Management, 40, 317-328. 10.1006/jema.1994.1025

## 1 Value of the Data

• These data provide an overview of bird communities and Red Squirrel occurrences in the mountain pine forests of the central Pyrenees in the late 1980s.
• Additionally, these data indicate the altitude, cover of open areas, stones, boulders and vegetation at different heights, canopy height and number of dead trees, which characterize the surveyed habitats, together with forest patch area and isolation.
• These data can be valuable for researchers studying bird communities, biodiversity, Red Squirrel occurrence, and typical mountain species such as Capercaillie, and for conservation actors.
• These data should be of interest to researchers concerned with the evolution of biodiversity or the abundance of species of interest since 1987 in mountainous areas, due to several drivers of change such as climate change or forest fragmentation. Each point count is marked on a map and new counts could easily be carried out at the same locations.
• These data should be useful for biogeographical comparisons with other mountain or lowland sites in terms of bird communities and squirrel occurrence.

## 2 Background

The extent and pattern of mountain pine forests are limited by geomorphology, altitude, fire and grazing. As a consequence, pine forests are fragmented into patches of different size, surrounded by a more or less rocky grassland and by heaths. Pine fragmentation in Néouvielle, a massif in the central French Pyrenees, represents a current situation in high altitude forests. Pine forests are sometimes connected with low-altitude forests, which harbour silver firs (Abies pectinata L.), beeches (Fagus sylvatica L.) and larch (Larix decidua L.).

The surveys carried out give a good picture of the bird communities and squirrel occurrence in the fragmented mountain pine forests of the high Pyrenees at the end of the 1980s. The climate was colder then, and our data could be used as a reference for studies on the evolution of bird communities and squirrel occurrence as a result of climate change that has taken place in recent decades.

Interestingly, these data could be compared to those recorded in the early 1980s in mountain pine forests of the eastern Pyrenees [1].

## 3 Data Description

**Fig. 1:**
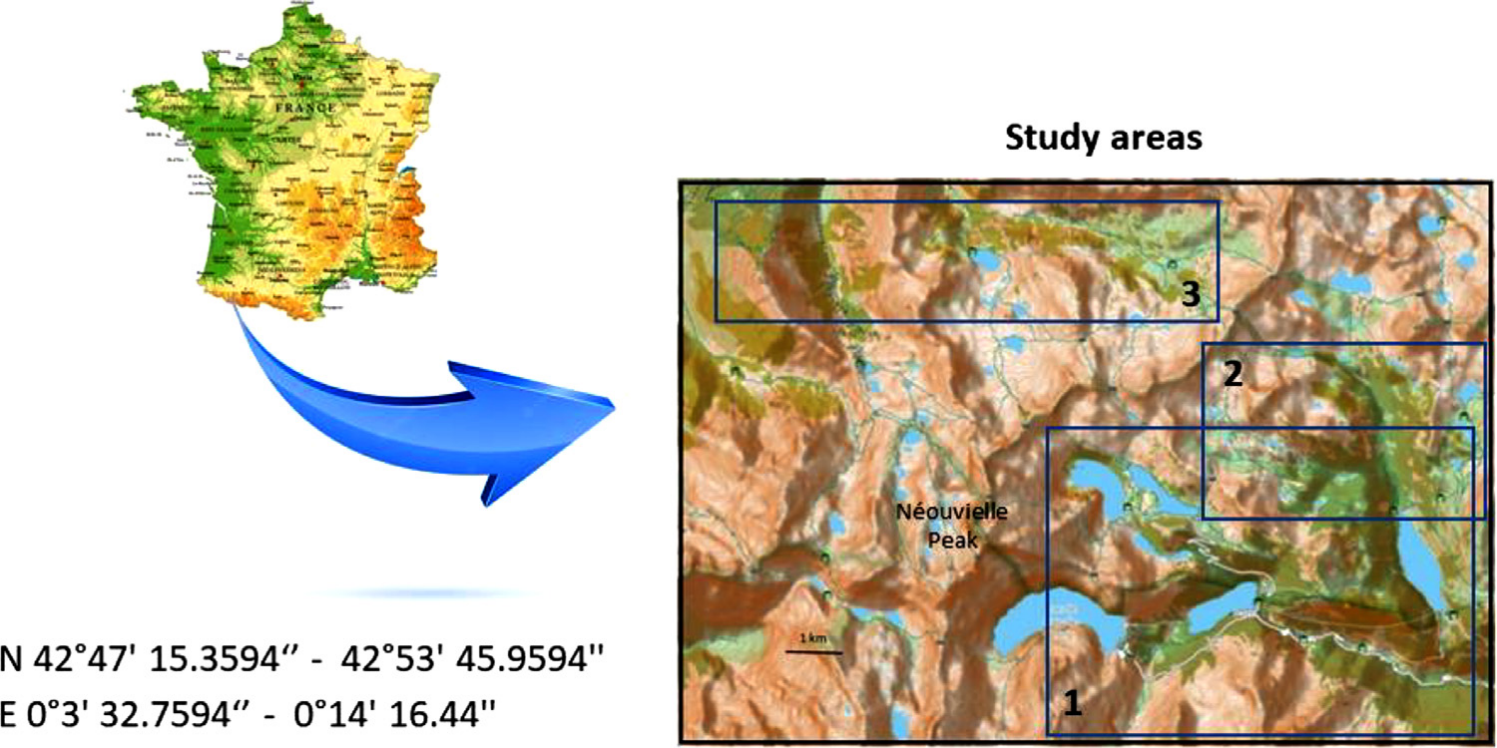
Location of the central Pyrenees and the three main study areas on the topographic map provided by openstreet map. The current forests are represented in green (this coverage may differ from that of 1987).

The dataset, which is available on Recherche Data Gouv [2], includes 197 records of Red Squirrel and breeding birds (“Neouvielle_signs_of_presence.tab” and “Neouvielle_birds.tab”) from mountain pine forests at altitudes between 1800 and 2400 m in the Néouvielle National Nature Reserve in the central French Pyrenees (Fig. 1 and “Neouvielle_maps.pdf”). A total of 16 birds’ species (14 by song and 2 by other signs of presence) were recorded in spring 1987 (Tables 1 and 2).

**Table 1:** Frequency of birds recorded using the point count technique (% of total point-counts).

Name	Frequency
Coal Tit (Periparus ater)	0.76
Crested Tit (Lophophanes cristatus)	0.27
Goldcrest (Regulus regulus)	0.36
Common Chaffinch (Fringilla coelebs)	0.66
Eurasian Wren (Troglodytes troglodytes)	0.02
Eurasian Treecreeper (Certhia familiaris)	0.04
Eurasian Jay (Garrulus glandarius)	0.02
Great Spotted Woodpecker (Dendrocopos major)	0.04
Mistle Thrush (Turdus viscivorus)	0.18
Yellowhammer (Serinus citrinella)	0.31
Ring Ouzel (Turdus torquatus)	0.07
Dunnock (Prunella modularis)	0.38
Grey Wagtail (Motacilla cinerea)	0.04
Tree Pipit (Anthus trivialis)	0.04

**Table 2:** Frequency of capercaillie droppings and pine cones consumed by birds and squirrels in 1986 (% of total point counts).

Name	Frequency
Capercaillie (Tetrao urogallus)	0.28
Common crossbill (Loxia curvirostra)	0.64
Great spotted woodpeckers (Dendrocopos major)	0.27
Red Squirrel (Sciurus vulgaris)	0.38

The altitude, cover of within-forest open areas, of stones, boulders and vegetation layers at different heights (<0.25 m, 0.25-0.5 m, 0.5-1 m, 1-2 m, 2-4 m, 4-8 m, 8-16 m, >16 m) were assessed within a 50m radius of the bird count site (Table 3). In addition, canopy height (m) and number of dead trees were recorded (“Neouvielle_habitats.tab”).

**Table 3:** Habitat and landscape variables estimated for each sampling point.

	Code	Meaning
Habitat (within the 50m point-count radius)		
	Altitude	Altitude (m)
	Open	Cover of within-forest open areas (%)
	Stones	Cover of stones (<0.5m in diameter) (%)
	Boulders	Cover of boulders (> 0.5m in diameter) (%)
	L1	Cover of vegetation layer <0.25 m (%)
	L2	Cover of vegetation layer 0.25-0.5 m (%)
	L3	Cover of vegetation layer 0.5-1 m (%)
	L4	Cover of vegetation layer 1-2 m (%)
	L5	Cover of vegetation layer 2-4 m (%)
	L6	Cover of vegetation layer 4-8 m (%)
	L7	Cover of vegetation layer 8-16 m (%)
	L8	Cover of vegetation layer >16 m (%)
	Canopy height	Canopy height (m)
	Number of dead trees	Number of dead trees
Landscape		
	A	Forest patch area (ha)
	DP	Distance to the nearest large pine stand (km)
	DLA	Distance to the nearest low-altitude forest (km)

The forests are fragmented into patches of varying sizes surrounded by rocky grasslands and heaths. Some pine forests are connected with lower altitude forests. For each record, the area of the forest stand containing the record and the distance to the nearest low-altitude forest were estimated. Forest patches of more than 50 ha were classified as large. Then, for records in forest stands smaller than 50 ha, the distance to the nearest large pine stand was estimated, otherwise it was set to zero (“Neouvielle_fragm.tab”).

The meaning of the rows and columns in the four files of the dataset is explained in a readme file (“Readme.pdf”).

## 4 Experimental Design, Materials and Methods

Birds were sampled using the point-count technique [3] within a limited distance of 50 m around the observer. Singing passerines and *picidae* were recorded for 20 min in spring mornings under fair weather conditions (no wind or rain). The bird species were identified by expert ornithologists (F.L. and M.G.).

Due to an exceptionally low number of pine cones in 1987, the Common Crossbill, a typical bird of these forests, was absent. The occurrence of pine cones consumed by crossbills in the previous year was noted on the ground within 50 m around the observer, as well as those consumed by woodpeckers and squirrels. Capercaillie, which are difficult to count using the point count technique, were recorded as droppings within 50 m of the observer.

**Fig. 2:**
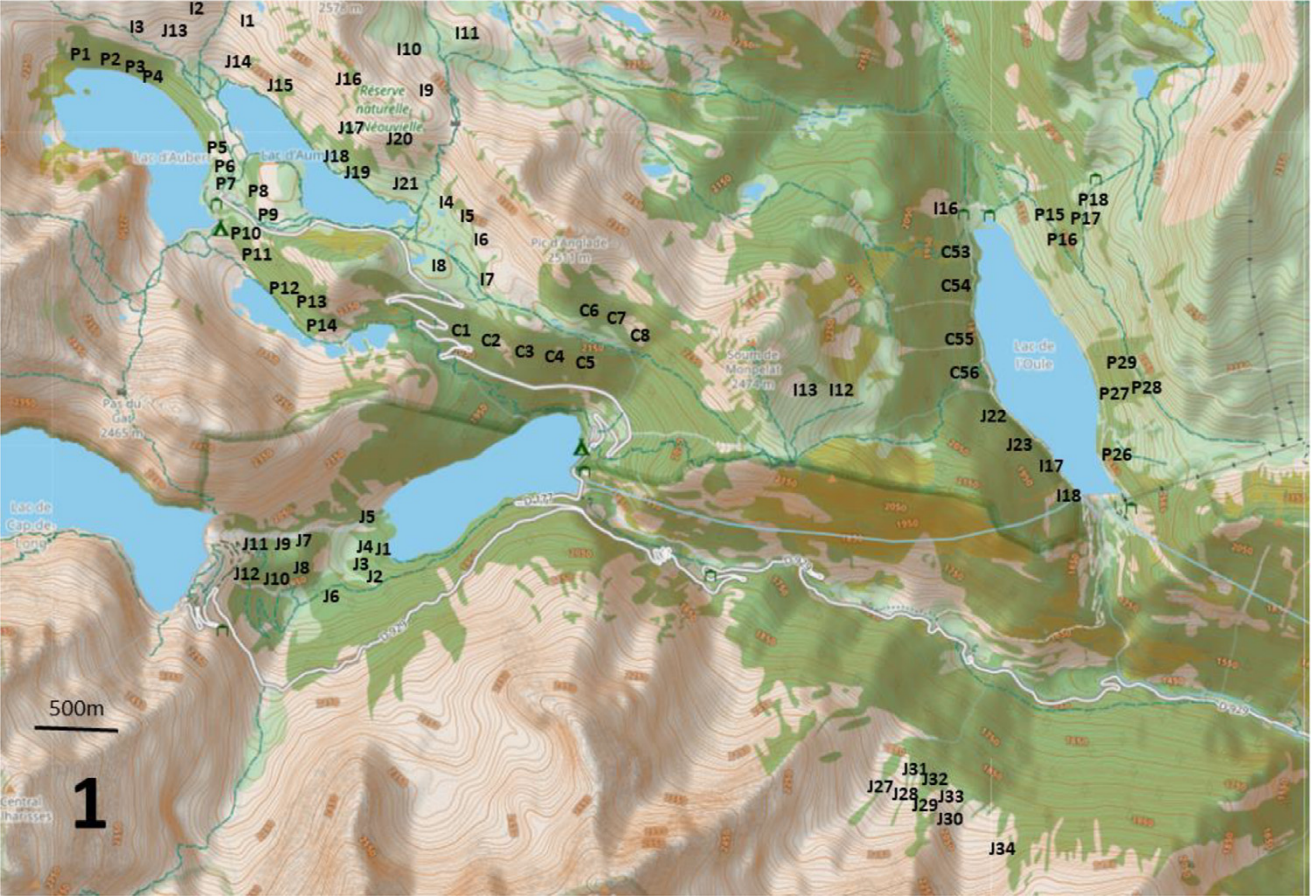
Location of the point-counts in the first study area.

**Fig. 3:**
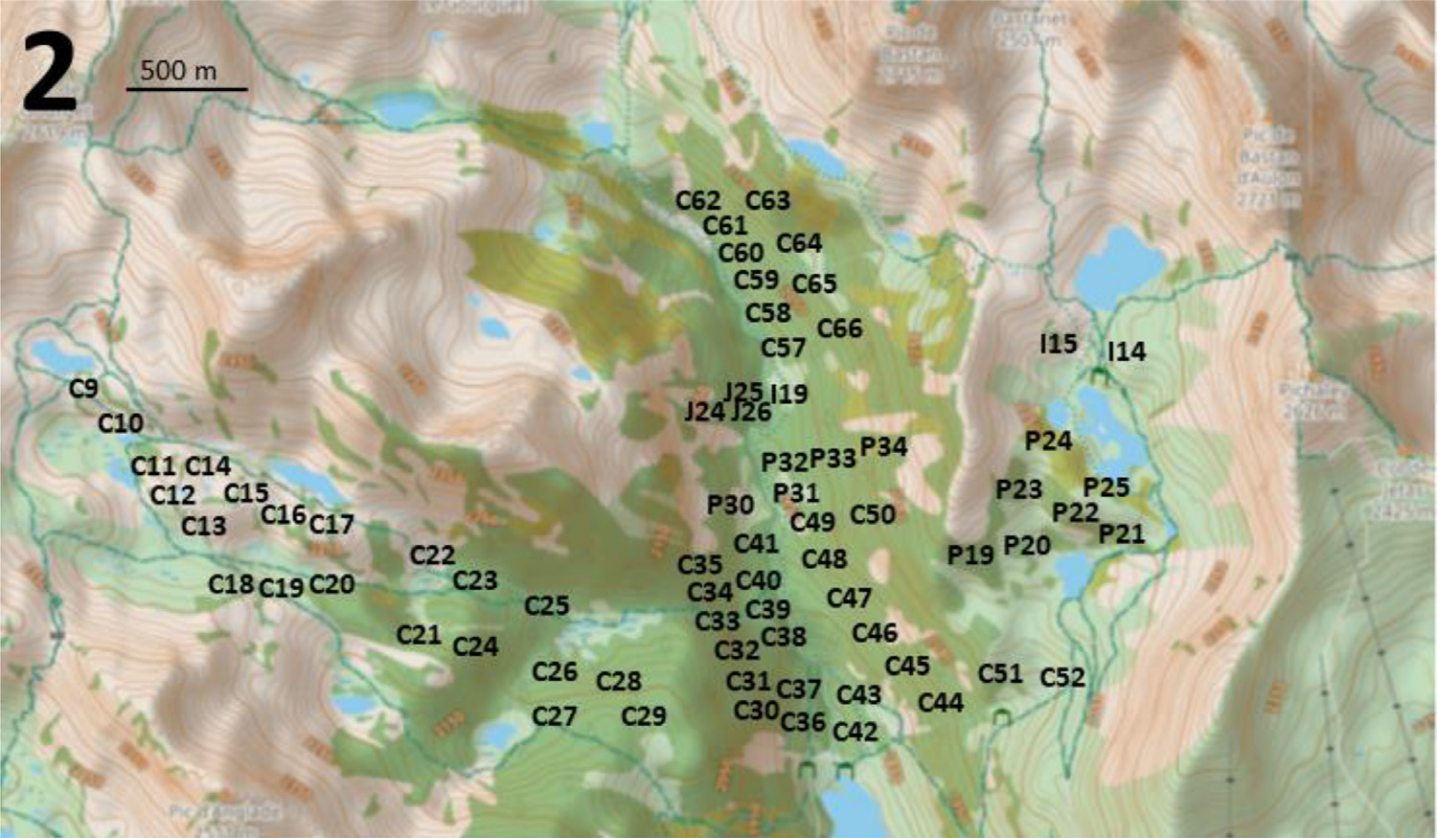
Location of the point-counts in the second study area.

**Fig. 4:**
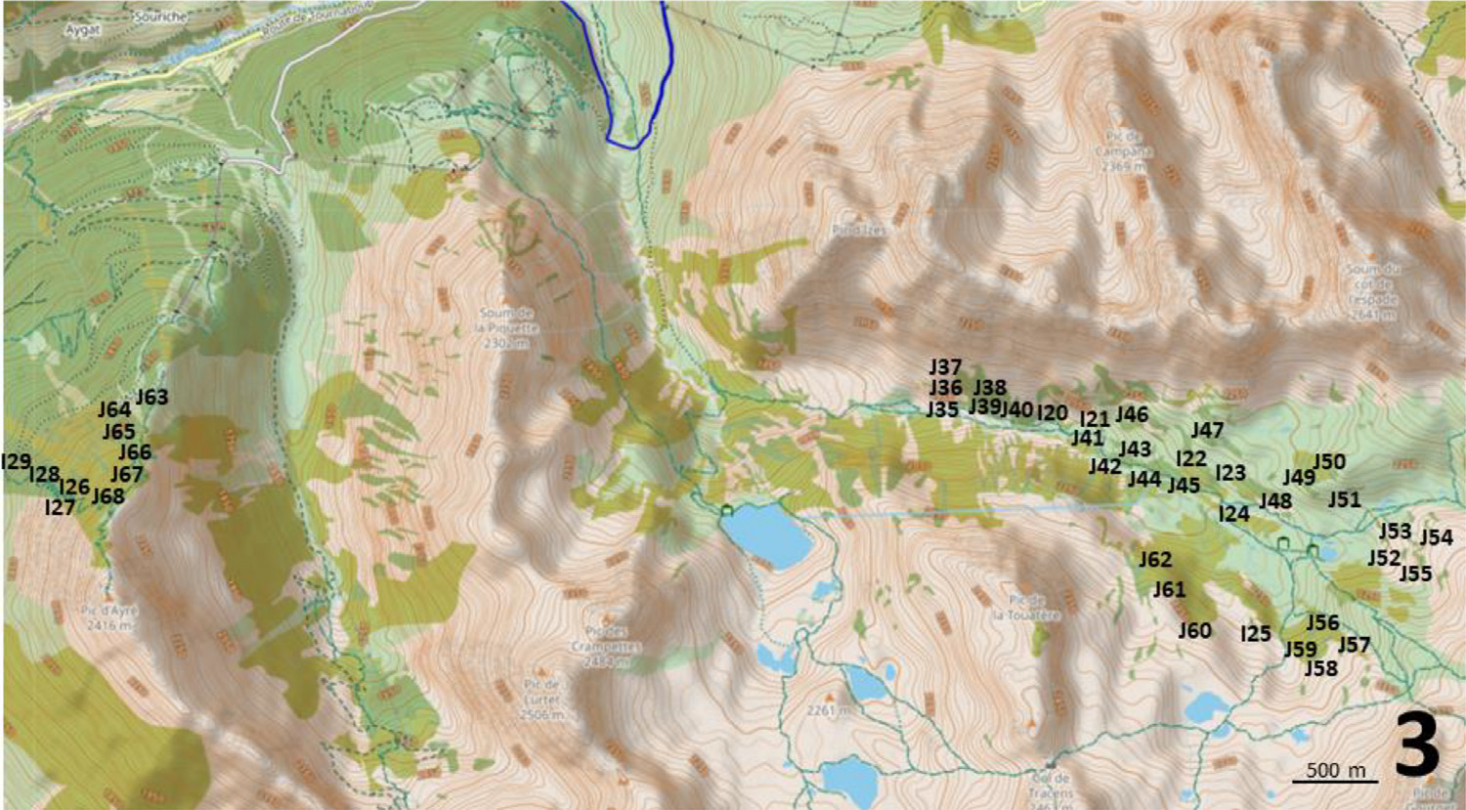
Location of the point-counts in the third study area.

Points-counts were located in three areas of the Néouvielle National Nature Reserve (Fig. 1). Their positions are indicated on Fig. 2, Fig. 3, Fig. 4. Habitat descriptors were evaluated within a 50-metre radius of the point-count site. Plant cover was estimated through comparison with reference drawings depicting imaginary cover levels of 5 %, 10 %, and so on [4]. The altitude was measured using an altimeter and the IGN map at a 1:25,000 scale.

The values of the landscape variables, describing both patch size and isolation from either large pine stands or low-altitude forest, were evaluated from 1/25,000 IGN maps and from 1/20,000 IGN aerial photographs*.*

## Limitations

Not applicable*.*

## Ethics Statement

The authors have read and follow the ethical requirements for publication in Data in Brief and confirm that the current work does not involve human subjects, animal experiments, or any data collected from social media platforms*.*

## CRediT authorship contribution statement

**Michel Génard:** Data curation, Writing – original draft. **Françoise Lescourret:** Data curation, Writing – original draft.

## Data Availability

Census of breeding birds in fragmented high-altitude mountain pine forests of the Central French Pyrenees in 1987 (Original data) (https://entrepot.recherche.data.gouv.fr/dataverse/inrae).
